# Clinicopathological characteristics and outcomes of rare histologic subtypes of gallbladder cancer over two decades: A population-based study

**DOI:** 10.1371/journal.pone.0198809

**Published:** 2018-06-11

**Authors:** Sandeep Samuel, Sarbajit Mukherjee, Nischala Ammannagari, Venkata K. Pokuri, Boris Kuvshinoff, Adrienne Groman, Charles M. LeVea, Renuka Iyer

**Affiliations:** 1 Department of Medicine, SUNY (State University of New York), Buffalo, New York, United States of America; 2 Division of Hematology-Oncology, University of Oklahoma Health Sciences Center, Oklahoma City, Oklahoma, United States of America; 3 Department of Medical Oncology, Roswell Park Cancer Institute, Buffalo, New York, United States of America; 4 Department of Surgical Oncology, Roswell Park Cancer Institute, Buffalo, New York, United States of America; 5 Department of Biostatistics/Bioinformatics, Roswell Park Cancer Institute, Buffalo, New York, United States of America; 6 Department of Pathology, Roswell Park Cancer Institute, Buffalo, New York, United States of America; University Hospital Llandough, UNITED KINGDOM

## Abstract

**Background:**

There is limited literature about the clinicopathological characteristics and outcomes of rare histologic variants of gallbladder cancer (GBC).

**Methods:**

Using SEER database, surgically managed GBC patients with microscopically confirmed adenocarcinoma, adenosquamous/squamous cell carcinoma and papillary carcinoma were identified from 1988 to 2009. Patients with second primary cancer and distant metastasis at presentation were excluded. The effect of clinicopathological variables on overall survival (OS) and disease specific survival (DSS) were analyzed using univariate and multivariate proportional hazards modeling. All associations were considered statistically significant at an alpha error of 0.01.

**Results:**

Out of 4738 cases, 217 adenosquamous/squamous (4.6%), 367 papillary (7.7%), and 4154 adenocarcinomas (87.7%) were identified. Median age was 72 years. Higher tumor grade (grade 2, 3, 4 versus grade 1), higher T stage (T2, T3, T4 versus T1), lymph node positivity (N1 versus N0) and adenosquamous/squamous histology (versus adenocarcinoma) had worse OS and DSS (p < .001). Papillary GBC had better OS and DSS than adenocarcinoma (HR = 0.7; p < .001). Radical surgery (versus simple cholecystectomy) had better OS (HR = 0.83, p = 0.002) in multivariate analysis. OS rates at 3 and 5 years were 0.56 and 0.44 for papillary, 0.3 and 0.22 for adenocarcinoma, and 0.14 and 0.12 for adenosquamous/squamous histology, while DSS rates at 3 and 5 years were 0.67 and 0.61 for papillary, 0.38 and 0.31 for adenocarcinoma, and 0.17 and 0.16 for adenosquamous/squamous subtypes respectively.

**Conclusion:**

Papillary GBC had better survival outcomes while adenosquamous/squamous GBC had worse survival outcomes compared to gallbladder adenocarcinoma.

## Introduction

Gall bladder cancer (GBC) is the most common biliary tract malignancy and the fifth most common gastrointestinal cancer [1, 2]. GBC and nearby large bile duct cancers accounted for an estimated 11,420 new cases and 3710 deaths in the United States in 2016. GBC has a dismal prognosis and majority of the cases are asymptomatic and are incidentally diagnosed during gall stone exploration or after cholecystectomy performed for a non-malignant indication[3]. Therefore, the index surgical procedure is often a simple resection of the gallbladder and a revision surgery is planned based on the staging results [4]. According to the National Comprehensive Cancer Network (NCCN) guidelines [2], a simple cholecystectomy is an adequate treatment for T1a tumors. While there is some controversy over T1b tumors [2], a complete surgical resection consisting of cholecystectomy with a limited hepatic resection and portal lymphadenectomy is the only curative treatment for T2 or greater tumors. It is performed either as an index procedure or as a revised procedure at a later date.

Adenocarcinoma is the most common histologic subtype in GBC, representing approximately 76–90% of cases (5). Among other GBC histologic subtypes, papillary tumors constitute 5–6%, while squamous and adenosquamous constitute 2–10% of cases [5, 6]. Due to the rarity of these histologic subtypes, current literature on the behavior and clinical outcomes of papillary and adenosquamous/squamous gall bladder cancer is limited to case reports or single institution studies [6–10]. The aim of this study was to identify the effects of tumor characteristics, clinicopathological variables and surgery on survival outcomes for these rare histologic variants in comparison to adenocarcinoma.

## Materials and methods

### Data source

Surveillance, Epidemiology, and End Results (SEER) Program database was used to identify the cohort of patients for this retrospective analysis. SEER database contains cancer specific data of approximately 26% of the Unites States population from 18 cancer registries and 14 geographically distinct regions. Specific de-identified data pertaining to demographics, tumor stage (TNM) and histologic grade, cancer directed surgery and radiation treatment is captured in the SEER database.

### Study population

The study cohort included patients diagnosed with gall bladder cancer from 1988 to 2009. Cases diagnosed prior to 1988 were not included as the type of cancer directed surgery is not specified in SEER database. The site specific ICD code of C.23.9 was used to identify gallbladder cancer patients. The histologic subtype of the tumor was identified using specific coding data inside the SEER database i.e. code 8140 represents adenocarcinoma, codes 8070, 8071, 8075, and 8560 represent all or predominant squamous histology (squamous and adenosquamous), and codes 8050, and 8260 represent all or predominant papillary histology (papillary carcinoma and papillary adenocarcinoma). All tumor stages except premalignant lesions (e.g. carcinoma in situ) and distant metastases were included in the study. Patients with a second primary cancer diagnosis and patients with cancer diagnosed at autopsy were excluded from the study. Patients who underwent a cancer directed surgery (index procedure) were identified in the study sample by comparing the diagnosis codes with surgery/procedure codes. The cancer directed surgery is either cholecystectomy (simple removal of gall bladder with or without regional lymph node dissection) or a radical surgical resection (removal of the gallbladder with partial or complete removal of surrounding structures, i.e. partial or total hepatic lobectomy with or without bile duct resection).

### Statistical analysis

Univariate associations between covariates and histology (adenocarcinoma, papillary carcinoma and adenosquamous/squamous carcinoma) were examined with the Kruskal-Wallis test for ordinal variables and the Pearson Chi-square test for categorical variables. Covariates included age, sex, race, tumor characteristics (T, N status, histologic grade, and tumor size,), type of surgery and radiation treatment. Univariate and multivariate Cox proportional hazards modeling results were used to assess the effect of histology and covariates on survival. Relative prognosis was summarized using estimates and 95% confidence limits for the hazard ratio (HR). Overall survival (OS), defined as the time (in months) from diagnosis to death from any cause, was the primary endpoint. Disease specific survival (DSS), defined as the time (in months) from diagnosis to death specifically from cancer, was the secondary endpoint. Patients dying from other causes were censored at date of death, and those alive were censored at the date of last follow up. Kaplan-Meier method was used to derive the OS and DSS. All associations were considered statistically significant at an alpha error of 0.01 (P value 0.01). All statistical analyses were performed using the SAS software version 9.2.

## Results

14,349 patients with GBC were identified between 1988-and 2009. Of these, 6004 patients belonged to the three histological subtypes of interest; adenocarcinoma (n = 5321; 88.6%), adenosquamous/squamous (n = 284; 4.7%) and papillary (n = 399; 6.6%). Among the adenosquamous/squamous GBC patients, 157 (55%) were adenosquamous, while most of the papillary GBC group (n = 382; 96%) included papillary adenocarcinoma.

Of the total 6004 cases of interest, 1266 (21.1%) patients had distant metastatic disease who were excluded from further analysis. Out of 4738 cases, there were 217 adenosquamous/squamous (4.6%), 367 papillary (7.7%), and 4154 adenocarcinoma (87.7%). Overall, 80% of the GBC population was white and 9% was black. Male to female ratio was 1:3. The mean age at diagnosis was 72 years. Table 1 shows the baseline characteristics of the three histologic subtypes. T3 and T4 status were more prevalent in adenosquamous/squamous GBC (52.5% and 11.5% respectively) compared to papillary (15.8% and 1.6%) and adenocarcinoma (39.5% and 5%) patients (p< 0.001). Also, adenosquamous/squamous GBC patients had worse histologic grade of tumor at presentation; grade 3 and grade 4 cancers in adenosquamous/squamous group constituted 38.7% and 1.8%, papillary group were 9.5% and 0% and adenocarcinoma group were 34.2% and 1.3% respectively (p<0.001). Regional and metastatic spread of disease was statistically more prevalent in adenosquamous/squamous GBC patients at the time of diagnosis. Lymph node involvement was lower in the papillary group (9.5% N1) compared to the adenosquamous/squamous (9.8% N1) and adenocarcinoma (21% N1) group of patients (p<0.001).

**Table 1:** Descriptive statistics by histology.

Characteristics		Papillary N = 367 (7.7%)	Adenosquamous/Squamous N = 217(4.6%)	Adenocarcinoma N = 4154 (87.7%)	Total N = 4738 (100%)	p value
Sex	Female	271 (73.8%)	154 (71.0%)	3,053 (73.5%)	3,478 (73.4%)	0.7
	Male	96 (26.2%)	63 (29%)	1,101 (26.5%)	1,260 (26.6%)	
Age, median		70 years	70 years	73 years	72 years	0.001
Race	White	265 (72.2%)	176 (81.1%)	3,325 (80.0%)	3,766 (79.5%)	0.007
	Black	39 (10.6%)	17 (7.8%)	354 (8.5%)	410 (8.7%)	
	Others	63 (17.2%)	24 (11.1%)	475 (11.4%)	562 (11.9%)	
T status	T1	200 (54.5%)	39 (18.0%)	1,052 (25.3%)	1,291 (27.2%)	<0.001
	T2	103 (28.1%)	39 (18.0%)	1,254 (30.2%)	1,396 (29.5%)	
	T3	58 (15.8%)	114 (52.5%)	1,640 (39.5%)	1,812 (38.2%)	
	T4	6 (1.6%)	25 (11.5%)	208 (5.0%)	239 (5.0%)	
Node status	N0	275 (74.9%)	123 (56.7%)	2,443 (58.8%)	2,841 (60.0%)	<0.001
	N1	35 (9.5%)	43 (19.8%)	874 (21.0%)	952 (20.1%)	
	NX	57 (15.5%)	51 (23.5%)	837 (20.1%)	945 (19.9%)	
SEER historic staging (excluding metstatic)	Localized	301 (82%)	91 (41.9%)	2,407 (57.9%)	2,799 (59.1%)	<0.001
	Regional	66 (18%)	126 (58.1%)	1,747 (42.1%)	1,939 (40.9%)	
Histologic grade	1	119 (32.4%)	8 (3.7%)	647 (15.6%)	774 (16.3%)	<0.001
	2	148 (40.3%)	83 (38.32%)	1,688 (40.6%)	1,919 (40.5%)	
	3	35 (9.5%)	84 (38.7%)	1,420 (34.62%)	1,539 (32.5%)	
	4	0 (0%)	4 (1.8%)	53 (1.3%)	57 (1.2%)	
Surgery	Simple resection	332 (90.5%)	178 (82.0%)	3,743 (90.1%)	4,253 (89.8%)	<0.001
	Radical surgical resection	35 (9.5%)	39 (18.0%)	411 (9.9%)	485 (10.2%)	
Radiation treatment	Yes	55 (15.0%)	51 (23.5%)	795 (19.1%)	901 (19.1%)	0.021
	No	307 (83.7%)	158 (72.8%)	3,280 (79.0%)	3,745 (79.0%)	
	Unknown	5 (1.4%)	8 (3.7%)	79 (1.9%)	92 (1.9%)	

Univariate and multivariate modeling for OS and DSS were performed to determine predictors of outcome with regards to various clinicopathological variables. The variables included in the model were T-status (T3, T2, T4 versus T1), N status (N0 versus N1), histologic grade of tumor (2, 3, 4 versus 1), size of tumor (>5cm versus <5cm), histology of the tumor (papillary, adenosquamous/squamous versus adenocarcinoma), index surgery (radical surgical resection versus simple resection) and radiation treatment (treatment versus no radiation treatment). Advanced T and N status, higher histologic grade, adenosquamous/squamous histology, and size >5cm were predictors of poor survival (see Tables 2 and 3) in both univariate and multivariate analysis. Papillary histology had better survival outcomes compared to adenosquamous/squamous and adenocarcinoma in both univariate and multivariate analysis for OS and DSS.

**Table 2:** Univariate and multivariate analysis model for overall survival.

Independent variables	Reference	Univariate model			Multivariate model		
		Hazard ratio	95% CI	p-value	Hazard ratio	95% CI	p-value
T status							
T2	T1	1.19	(1.08, 1.31)	< .001	1.16	(1.05, 1.27)	0.004
T3		2.47	(2.26, 2.69)	< .001	2.2	(2.01, 2.41)	< .001
T4		4.04	(3.48, 4.69)	< .001	3.53	(3.01, 4.14)	< .001
Histologic grade							
2	Grade 1	1.39	(1.25, 1.54)	< .001	1.26	(1.14, 1.40)	< .001
3		2.29	(2.06, 2.54)	< .001	1.81	(1.62, 2.02)	< .001
4		2.19	(1.64, 2.92)	< .001	1.86	(1.39, 2.49)	< .001
Size							
> = 5cm	Size < 5cm	1.4	(1.26, 1.56)	< .001	1.18	(1.06, 1.32)	0.003
Unknown		1.57	(1.42, 1.74)	< .001	1.37	(1.24, 1.52)	< .001
Node status							
N1	N0	1.53	(1.40, 1.66)	< .001	1.28	(1.17, 1.40)	< .001
Nx		1.48	(1.36, 1.61)	< .001	1.26	(1.16, 1.37)	< .001
Histology							
Papillary	Adenocarcinoma	0.52	(0.45, 0.60)	< .001	0.74	(0.64, 0.86)	< .001
Adenosquamous/Squamous		1.58	(1.36, 1.84)	< .001	1.29	(1.11, 1.50)	0.001
Surgery							
Radical surgical resection							
	Simple resection	1.07	(0.96, 1.19)	0.246	0.83	(0.74, 0.93)	0.002
Radiation							
No radiation	Yes	1.12	(1.03, 1.22)	0.006	1.49	(1.36, 1.62)	< .001
Unknown		1.61	(1.26, 2.04)	< .001	1.93	(1.52, 2.46)	< .001

**Table 3:** Univariate and multivariate analysis model for disease specific survival.

Independent variables	Reference	Univariate model			Multivariate model		
		Hazard ratio	95% CI	p-value	Hazard ratio	95% CI	p-value
T status							
T2	T1	1.37	(1.22, 1.54)	< .001	1.29	(1.14, 1.46)	< .001
T3		3.25	(2.93, 3.61)	< .001	2.72	(2.43, 3.04)	< .001
T4		5.62	(4.76, 6.64)	< .001	4.45	(3.73, 5.31)	< .001
Histologic grade							
2	Grade 1	1.61	(1.42, 1.83)	< .001	1.38	(1.17, 1.66)	< .001
3		2.84	(2.50, 3.23)	< .001	2.04	(1.79, 2.33)	< .001
4		2.89	(2.11, 3.95)	< .001	2.22	(1.62, 3.05)	< .001
Size							
> = 5cm	Size < 5cm	1.53	(1.35, 1.73)	< .001	1.23	(1.08, 1.39)	0.002
Unknown		1.7	(1.51, 1.91)	< .001	1.45	(1.28, 1.63)	< .001
Node status							
N1	N0	1.78	(1.62, 1.95)	< .001	1.36	(1.23, 1.50)	< .001
Nx		1.63	(1.48, 1.79)	< .001	1.34	(1.22 1.48)	< .001
Histology							
Papillary	Adenocarcinoma	0.4	(0.33, 0.48)	< .001	0.63	(0.52, 0.76)	< .001
Adenosquamous/Squamous		1.83	(1.56, 2.15)	< .001	1.43	(1.21, 1.68)	< .001
Surgery							
Radical surgical resection							
	Simple resection	1.19	(1.06, 1.34)	0.005	0.87	(0.76, 0.98)	0.025
Radiation							
No radiation	Yes	1.16	(1.07, 1.26)	< .001	1.47	(1.35, 1.61)	< .001
Unknown		1.5	(1.19, 1.90)	< .001	1.76	(1.39, 2.23)	< .001

Radical surgical resection as index surgery predicted poor outcome compared to simple resection in univariate analysis for DSS (HR 1.19; 95% CI 1.06, 1.34, p = 0.005) but not OS (HR 1.07; 95% CI 0.96, 1.19, p = 0.25). However, after adjustment for tumor variables in the multivariate model, radical surgical resection predicted better survival compared to simple resection among the three histologies; HR for OS of 0.83, 95% CI 0.74–0.93, p = 0.002) and HR for DSS of 0.87, 95% CI 0.76–0.98, p = 0.025). Patients who did not receive radiation had poor survival outcomes compared to those who received radiation, with a HR for OS of 1.49 (95% CI 1.36, 1.62. p <0.001) and HR for DSS of 1.47 (95% CI 1.35, 1.61, p<0.001). The interaction between histologic type and radiation was not a significant predictor of survival.

**Fig 1:**
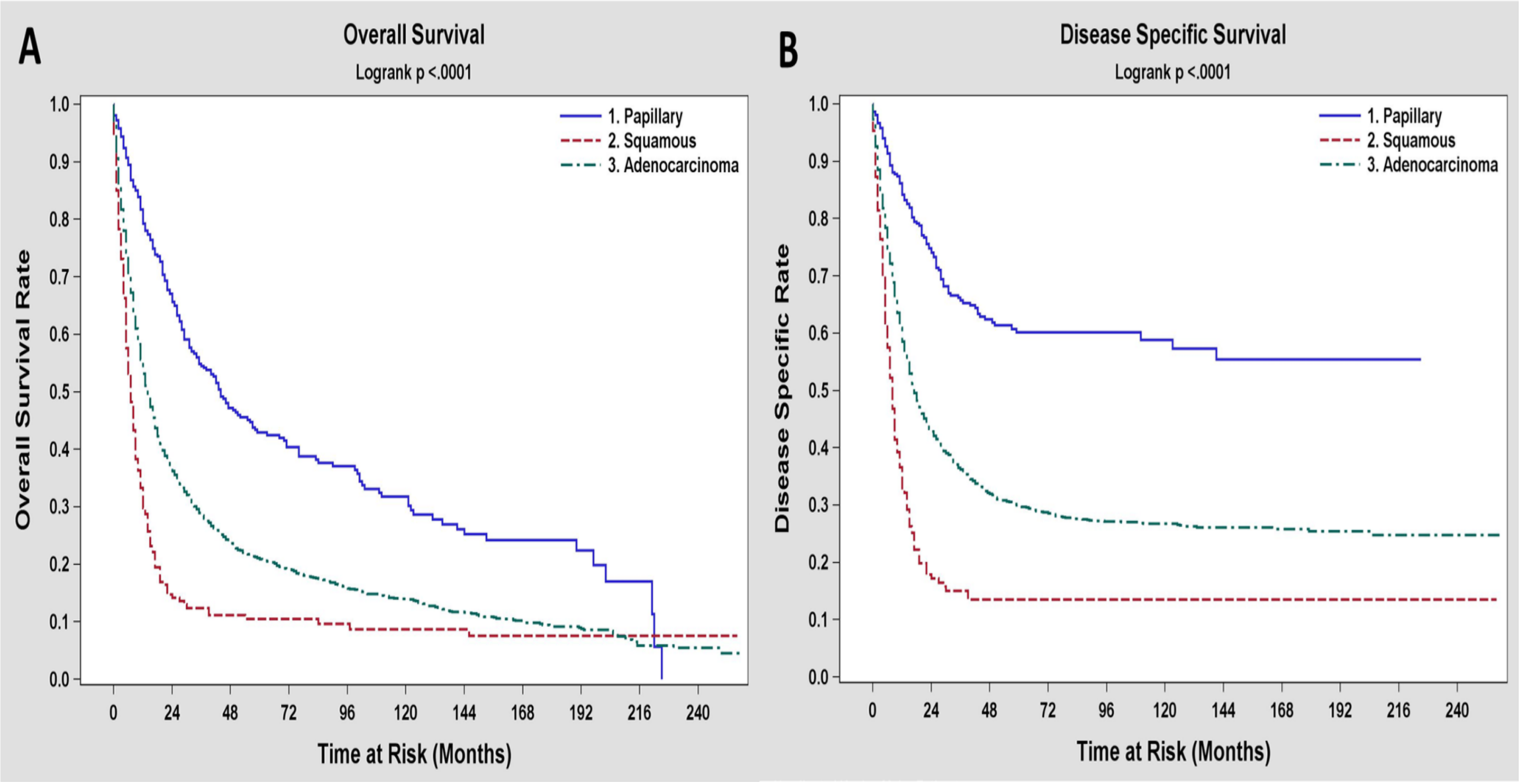
Kaplan-Meier curves for overall survival (A) and disease specific survival (B) based on the three histologic types of gallbladder carcinoma.

**Fig 2:**
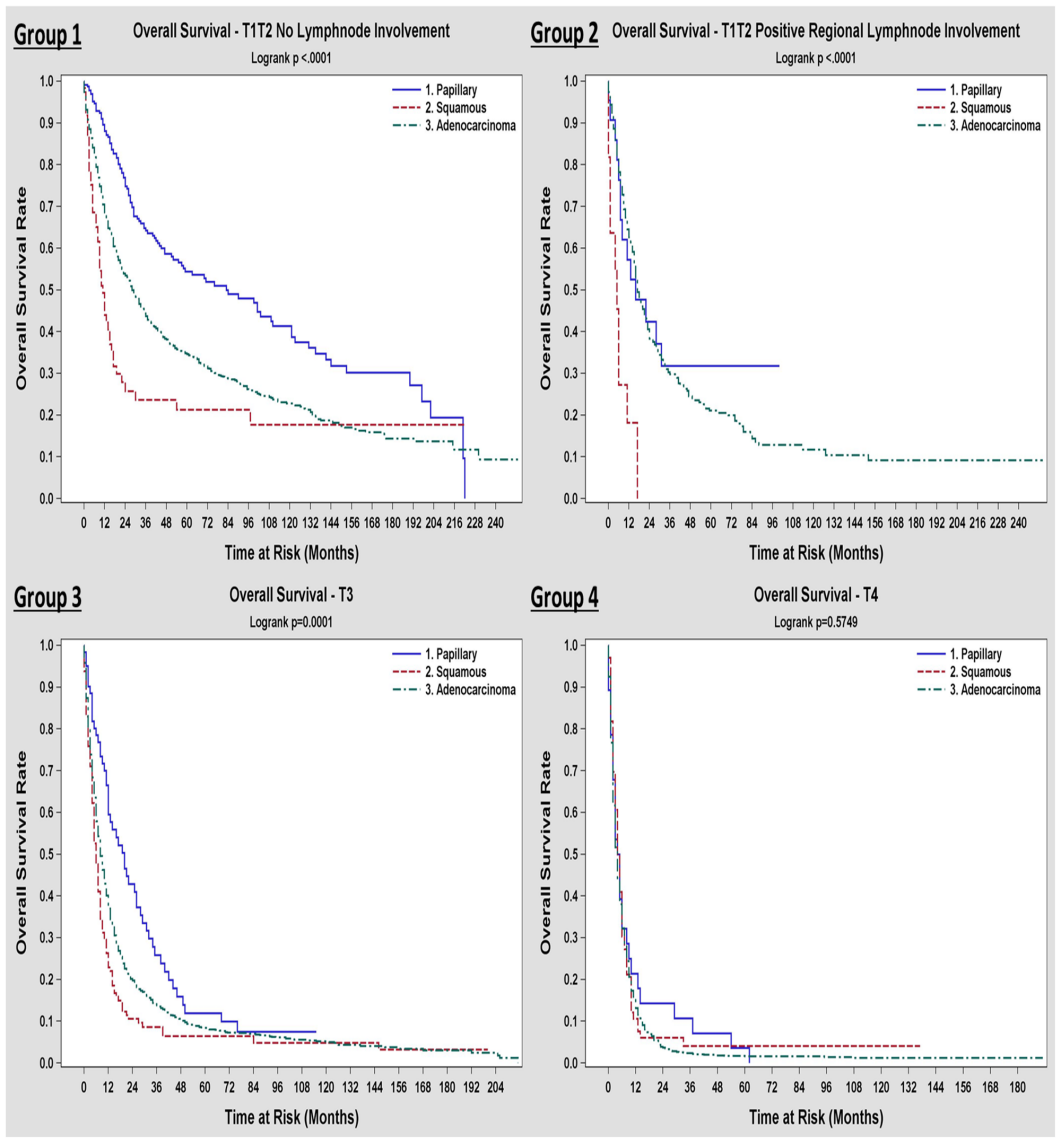
Kaplan-Meier overall survival curves among the three histologic types of gallbladder carcinoma based on the American Joint Committee on cancer staging groups [31].

The 3 and 5-year survival rates for the three different histologic types depicted in Table 4, suggesting better outcomes for papillary and worse outcomes for adenosquamous/squamous subtypes compared to adenocarcinoma. At the time of the analysis, 25.9% of the patients were alive at a median follow up time of 81 months. Unadjusted median overall survival was 15 months. Papillary GBC patients had better median OS (44 months), while the adenosquamous/squamous GBC patients had the worst outcome, with a median survival of 7 months and 5-year survival rate of 12%. Figs 1 and 2 show the Kaplan-Meier plots of OS and DSS for all the patients based on the three histologic types respectively. In our study, we did not see different outcomes across histology for T4 tumors. However when we looked at patients with earlier T-status with or without LN involvement, papillary histology had a better survival compared to adenocarcinoma and adenosquamous/squamous histology had the worst outcome (Fig 2). 3-year and 5-year survival as well as median survival data for different T and N-status across histology, is presented in S1 Table.

**Table 4:** Overall survival and disease-specific survival rates.

Survival	Papillary	Adenosqouamous/Squamous	Adenocarcinoma
Overall Survival			
3-year survival rate	0.56	0.14	0.30
5-year survival rate	0.44	0.12	0.22
Median overall survival (months)	44	7	15
Disease specific survival			
3-year survival rate	0.67	0.17	0.38
5-year survival rate	0.61	0.16	0.31

## Discussion

The rarity of adenosquamous/squamous and papillary histologic types of GBC in the general population precludes the description of their clinicopathological characteristics, survival outcomes and treatment responses [10, 11]. A national cancer registry like SEER database samples tumor data from approximately 26 percent of the US population and is an ideal tool for quality and outcome studies on rare cancer histology. To the best of our knowledge, our study is the largest in showing the clinicopathological characteristics and survival outcomes of the papillary, adenosquamous/squamous variants of GBC and comparing them with pure adenocarcinoma of gallbladder.

Squamous histology in gallbladder cancer could arise from pre-existing squamous metaplasia or squamous differentiation of the adenocarcinoma cells [11, 12]. The clinical outcomes of adenosquamous/squamous GBC were compared with those of gallbladder adenocarcinoma in prior small retrospective studies with discordant results, however, majority of them favored poor prognosis of adenosquamous/squamous GBC. Chan et al from Taiwan compared 14 adenosquamous/squamous GBC cases with adenocarcinoma controls and reported slightly better 3-year and 5-year survival rates for adenosquamous/squamous GBC [7]. Another retrospective study from Korea showed significant poorer 1-year survival in 16 adenosquamous/squamous GBC cases compared to adenocarcinoma controls (18.8% vs. 87.3%, P < 0.001)[13]. Two retrospective studies from Chile and China compared 34 cases of adenosquamous/squamous GBC each with adenocarcinoma [10, 11]; these studies identified a poor median OS for adenosquamous/squamous histology compared to adenocarcinoma that was statistically significant (p<0.05). Our study with a larger cohort of patients (n = 4738) confirmed poor survival outcomes for adenosquamous/squamous GBC compared to adenocarcinoma (median OS of 7 months versus 15 months; p < 0.001). Higher T resulting in poor outcomes for adenosquamous/squamous GBC patients and these features were consistent in prior smaller studies [10, 14]. Thus, adenosquamous/squamous GBC tend to have localized bulky growth with a propensity to infiltrate adjacent organs. In addition, several studies reported higher proliferative rate and lower lymphatic spread with adenosquamous/squamous GBC [14–16]. The risk of N1 disease was lower for adenosquamous/squamous GBC in our study compared to adenocarcinoma (19.8% versus 21%, p < 0.001).

Despite lower lymphatic spread of adenosquamous/squamous GBC, the features of higher T status at presentation and the tendency to infiltrate adjacent organs can pose a substantial challenge for the surgeons in obtaining tumor free margins at resection (R0), attaining which was shown to improve the prognosis and outcomes [13,17].

Pure papillary GBC is extremely rare with only 17 patients identified in our study. Majority of the papillary group of patients had predominant papillary differentiation within adenocarcinoma (papillary adenocarcinoma). The hypothesis that papillary adenocarcinomas originate from papillary adenomas might not be true, as some studies proved that this pathway plays only a minor role in GBC as opposed to the dysplasia-carcinoma pathway [18, 19]. A recent retrospective study by Cariati et al identified 100% association of pancreatobiliary reflux and C-Ki-ras point mutations with papillary adenocarcinoma of gallbladder [20]. In regards to prognosis, earlier studies suggested better survival outcomes with papillary GBC. Albores-Saavedra et al studied survival estimates of invasive papillary GBC retrospectively using SEER database and reported better survival rates (52% versus <10%) for those confined to the gallbladder wall, compared to those spread to the lymph nodes [21]. Patients with papillary histology in our study had significantly better survival outcomes (median OS of 44 months; 5 -year survival rate of 44%) compared to both adenocarcinoma and adenosquamous/squamous GBC. Papillary GBC tend to be of low histologic grade (approximately 73% with grade 1 and 2 disease in our study), but present as bigger 40% were ≥ 5cm) exophytic growths. Their delayed invasion of gallbladder wall results in much earlier stages at presentation (approximately 82% with T1/T2, 8% only with N1 in our study) compared to adenosquamous/squamous and adenocarcinomas. Recently, Wan et al from China reported similar clinicopathological features and improved 1-year, 3-year and 5- year survival rates for papillary adenocarcinomas as opposed to adenocarcinoma [22].

Surgery is considered the primary modality of treatment for patients diagnosed with localized GBC. Apart from tumor stage and biology, the extent of surgical resection has strong correlation with survival, as evident from several studies showing greater survival with radical resection in early stage GBC [23, 24]. The current NCCN guidelines and expert consensus recommend simple cholecystectomy for T1a disease, while extended surgery is associated with improved survival in T2 or greater tumors [2, 25]. In our study, compared to simple cholecystectomy, radical surgical resection as the index procedure showed improved OS and DSS in multivariate analysis, after adjustment for tumor characteristics and histology. While approximately 73% of our study population had ≥ T2 disease, only 10.2% had radical resection as the index surgery. A study by Mayo et al analyzed SEER data of surgically managed gallbladder adenocarcinoma patients over 15 years [26]. They reported a slightly higher rate of radical resection (13%) than our study as they linked the SEER data with Medicare claims data, resulting in more accurate capture of all surgical procedures. These figures suggest dramatic underutilization of radical surgery for early stage GBC. However, it should be acknowledged that SEER data lists only the index surgical procedure and it is very likely that a proportion of patients who underwent simple resection as index procedure could have had a more extensive revision surgery later on.

Certain other limitations of our study include absence of data on chemotherapy and performance status of the patients at diagnosis. Changes in clinical outcomes attributable to these factors are therefore not available. Attaining microscopic negative margins (R0 resection), aside from the extent of surgery, is the key determinant of surgical outcomes in early GBC [17, 27–30]. Information on the surgical margin status was not available from the SEER data in our study. Although radical/extended surgery showed better survival outcomes on multivariate analysis compared to simple cholecystectomy in the whole cohort, the survival analysis comparing these two surgical procedures among the different T and N status of papillary and adenosquamous/squamous GBC was not possible due to the very low number of patients reported to have extended surgery in each of these subgroups.

In summary, GBC is an aggressive malignancy with dismal outcome. Papillary and adenosquamous/squamous histologic variants of GBC are rare and differ from gallbladder adenocarcinoma in their clinicopathological characteristics. Most of the earlier studies in the literature reporting the outcomes of these rare histologies are very small in sample size and represented Asian population. Our study is unique in that it is the largest study to review and compare the clinicopathological features and survival outcomes of these rare histological variants of GBC with gallbladder adenocarcinoma in US population over a period of 21 years. We found that papillary GBC has the best survival outcomes following surgery, while adenosquamous/squamous GBC had the worst outcome in our large population based analysis. Differences in tissue invasion and lymph node spread among these different histologic types of GBC are important to consider while planning curative surgery. Based on our data, one may take a cautious approach to radical surgery in GB cancer with squamous/adenosquamous histology, especially in elderly or frail patients since the outcome is poor. In this era of precision medicine, studies are needed to analyze molecular and genetic mechanisms contributing to the differences in the behavior of these histologic variants, which could identify potential biomarkers predicting response to adjuvant therapies. Finally, clinicians should focus on enrolling GB cancer patients with rare histology in clinical trials that use novel therapies until new data becomes available.

## Supporting information

S1 Table Survival estimates based on TN staging. (XLSX)
